# Supplementary material for: Genome Characterization of Carbapenem-Resistant Hypervirulent Klebsiella pneumoniae Strains, Carrying Hybrid Resistance-Virulence IncHI1B/FIB Plasmids, Isolated from an Egyptian Pediatric ICU
Source: Microorganisms. 2025 May 1;13(5):1058. doi: 10.3390/microorganisms13051058 (PMC12114269; doi:10.3390/microorganisms13051058)

Genome Characterization of Carbapenem Resistant Hypervirulent *Klebsiella pneumoniae* Strains, Carrying Hybrid Resistance-Virulence IncHI1B/FIB Plasmids, Isolated from an Egyptian Pediatric ICU

Supplementary Material

Heba A. Hammad [1], Radwa Abdelwahab [1], Douglas F. Browning [2,*] and Sherine A. Aly [1,*]

[1] Department of Medical Microbiology and Immunology, Faculty of Medicine, Assiut University,
Assiut 71515, Egypt; hebaali@aun.edu.eg (H.A.H.); radwa.wahab418@gmail.com (R.A.)

[2] College of Health & Life Sciences, Aston University, Aston Triangle, Birmingham B4 7ET, UK

* Correspondence: d.browning@aston.ac.uk (D.F.B.);
s-aly71@windowslive.com or s-aly71@aun.edu.eg (S.A.A.)

Supplementary Figure legends

Supplementary Figure S1. Analysis of the IncHI1B/FIB hybrid plasmid carried by *K. pneumoniae* strains K22 and K45. **A)** The panel shows the alignment of *K. pneumoniae* plasmid pKP-1PI_HIB-FIB (CP071028.1) (also known as pVIR-147Tu [1]) with contigs from K45 (contig 12) and K22 (contig 23), which carry the IncHI1B (pNDM-Mar) replicon. **B)** The panel shows the alignment of pKP-1PI_HIB-FIB with contigs from K45 (contig 15) and K22 (contig 25), which carry the IncFIB (pNDM-Mar) replicon. **C)** The panel shows the alignment of pKP-1PI_HIB-FIB with contig 10 from the draft genome of K45, which carries various *K. pneumoniae* virulence determinants. In each panel, comparison was carried out using the Artemis Comparison Tool (ACT) [2] and alignment is shown by red and blue banding. Blue banding indicates that the sequences are inverted with respect to each other. Selected features that are shared between sequences are indicated.

Supplementary Figure S2. Comparison of the related IncL and IncM1 plasmid replicons carried by *K. pneumoniae* strains K22 and K45. The panel shows the alignment of the contigs carrying the IncL and IncM1 replicons from K22 (contig 30) and K45 (contig 14), respectively. Comparison was carried out using ACT [2]. Alignment is shown by blue banding, which indicates that the DNA sequence of contig 14 (K45) has been inverted to maintain the alignment with contig 30 (K22). Selected features, including the IncL and IncM1 replicons, have been labelled.

Supplementary Figure S3. Analysis of the IncL and IncM1 plasmid replicons carried by *K. pneumoniae* strains K22 and K45. **A)** The panel shows the alignment of *K. pneumoniae* plasmid pDT1 (CP019078.1) [3] with contig 30 from K22, which carries the IncL replicon. **B)** The panel shows the alignment of *Proteus mirabilis* plasmid pOXA48-Pm (KP025948.1) [4] with contig 14 from the draft genome of K45, which carries the IncM1 plasmid replicon. In each panel, comparison was carried out using ACT [2] and the alignment is shown by red and blue banding. Blue banding indicates that the DNA sequences have been inverted with respect to each other. Selected features, including the IncL and IncM1 replicons, have been indicated.

Supplementary Figure S4. Analysis of the $bla_{\text{NDM-1}}$ carrying plasmid from *K. pneumoniae* strain K22. **A)** The panel shows the alignment of *K. michiganensis* plasmid pK518_NDM1 (CP023187.1) [5] with contig 34 from K22, which carries the $bla_{\text{NDM-1}}$ carbapenemase gene. **B)** The panel shows the alignment of *K. michiganensis* plasmid pK518_NDM1 (CP023187.1) [5] with contig 27 from the K22 draft genome, which carries the IncFII(YP) plasmid replicon. In each panel, comparison was carried out using ACT [2] and the alignment is shown by red and blue banding. Blue banding indicates that the DNA sequences have been

inverted with respect to each other to maintain the alignment. Selected features have been indicated.

Supplementary Figure S5. Analysis of the IncFIB(pQil) and Col440II plasmid replicons carried by *K. pneumoniae* isolate K22. **A)** The panel shows the comparison of *K. pneumoniae* MGH 78578 plasmid pKPN4 (CP000649.1) [6] with *K. pneumoniae* K22 contig 38 (which carries the IncFIB(pQil) replicon) as well as the draft genome of K22, using ProkSee [7]. **B)** The panel shows the comparison of *K. pneumoniae* plasmid p2247421-T20-MAC_5 (CP086428.1) with *K. pneumoniae* K22 contig 44 (which carries the Col440II replicon) and the K22 draft genome, using ProkSee [7]. In both panels, the two outer rings display the genes/ CDS of each plasmid with selected features indicated.

Supplementary Figure S6. Analysis of the Col(pHAD28) and Col440I plasmid replicons carried by *K. pneumoniae* isolate K45. **A)** The panel shows the comparison of *K. pneumoniae* plasmid pKpVSI78_4 (CP118042.1) [8] with *K. pneumoniae* K45 contig 25 (which carries the Col(pHAD28) replicon) and the K45 draft genome, using ProkSee [7]. **B)** The panel shows the comparison of *K. pneumoniae* MGH 78578 plasmid pKPN6 (CP000651.1) [6] with *K. pneumoniae* K45 contig 27 (which carries the Col440I replicon) as well as the draft genome of K45, using ProkSee [7]. In both panels, the two outer rings display the genes/ CDS of each plasmid and selected features indicated.

Supplementary Figure S7. Analysis of *K. pneumoniae* strain K22 (EGY22) from Alexandria. The figure shows the comparison of **A)** the draft genome sequence of *K. pneumoniae* strain K22 from Alexandria (also known as isolate 678/0017 and EGY22) (DAMSAJ010000000), **B)** plasmid pEGY22_CTX_15 (ON261191.1) and **C)** plasmid pEGY22_CTX_14 (ON261190.1) [9] with the genome sequences of K22 and K45 from this study, as well as pKP-12PI_HIB-FIB (CP072918) (also known as pVIR-147Tu.12PI [1]) and pDT1 (CP019078.1) [3], using ProkSee [7]

Supplementary Figure S8. Analysis of hybrid *K. pneumoniae* AMR/ virulence plasmids. The figure shows the comparison of *K. pneumoniae* plasmids: **A)** pKpvST147L (CM007852.1) isolated in the UK in 2016 [10], **B)** p4313/18_1 **(**ON081621.1) isolated in Poland in 2018, **C)** pKpvST147B (CP040726.1) isolated in the UK in 2019 [11], **D)** phvKpST147_NDM-1_2566 **(**MW911671.1) isolated in Russia in 2019 [12], and **E)** pPAPIvir (CP084985.1) isolated in Italy in 2020 [13]. The sequence of each plasmid is compared with the draft genomes of *K. pneumoniae* K22 and K45 and the sequence of pKP-12PI_HIB-FIB (CP072918) (also known as pVIR-147Tu.12PI [1]), using ProkSee [7]. The green, brown and purple rings depict the BLAST results when the sequences of K22, K45 and pKP-12PI_HIB-FIB are compared with each plasmid.

.

Supplementary References

1. Di Pilato V, Henrici De Angelis L, Aiezza N, Baccani I, Niccolai C, Parisio EM, Giordano C, Camarlinghi G, Barnini S, Forni S, Righi L, Mechi MT, Giani T, Antonelli A, and Rossolini GM. (2022) Resistome and virulome accretion in an NDM-1-producing ST147 sublineage of *Klebsiella pneumoniae* associated with an outbreak in Tuscany, Italy: a genotypic and phenotypic characterisation. Lancet Microbe 3(3): e224-e234. 10.1016/s2666-5247(21)00268-8.
2. Carver TJ, Rutherford KM, Berriman M, Rajandream MA, Barrell BG, and Parkhill J. (2005) ACT: the Artemis Comparison Tool. Bioinformatics 21(16): 3422-3. 10.1093/bioinformatics/bti553.
3. Both A, Büttner H, Huang J, Perbandt M, Belmar Campos C, Christner M, Maurer FP, Kluge S, König C, Aepfelbacher M, Wichmann D, and Rohde H. (2017) Emergence of ceftazidime/avibactam non-susceptibility in an MDR *Klebsiella pneumoniae* isolate. J Antimicrob Chemother 72(9): 2483-2488. 10.1093/jac/dkx179.
4. Chen L, Al Laham N, Chavda KD, Mediavilla JR, Jacobs MR, Bonomo RA, and Kreiswirth BN. (2015) First report of an OXA-48-producing multidrug-resistant *Proteus mirabilis* strain from Gaza, Palestine. Antimicrob Agents Chemother 59(7): 4305-7. 10.1128/aac.00565-15.
5. Zheng B, Xu H, Yu X, Lv T, Jiang X, Cheng H, Zhang J, Chen Y, Huang C, and Xiao Y. (2018) Identification and genomic characterization of a KPC-2-, NDM-1- and NDM-5-producing *Klebsiella michiganensis* isolate. J Antimicrob Chemother 73(2): 536-538. 10.1093/jac/dkx415.
6. McClelland M, Sanderson KE, Spieth J, Clifton SW, Latreille P, Courtney L, Porwollik S, Ali J, Dante M, Du F, Hou S, Layman D, Leonard S, Nguyen C, Scott K, Holmes A, Grewal N, Mulvaney E, Ryan E, Sun H, Florea L, Miller W, Stoneking T, Nhan M, Waterston R, and Wilson RK. (2001) Complete genome sequence of *Salmonella enterica* serovar Typhimurium LT2. Nature 413(6858): 852-6. 10.1038/35101614.
7. Grant JR, Enns E, Marinier E, Mandal A, Herman EK, Chen CY, Graham M, Van Domselaar G, and Stothard P. (2023) Proksee: in-depth characterization and visualization of bacterial genomes. Nucleic Acids Res 51(W1): W484-w492. 10.1093/nar/gkad326.
8. Whidbey C, Konopaski AN, Teshome R, Dhaliwal S, Peters MQ, Mejia ME, Ballard MB, and Patras KA. (2023) Complete Genome Sequences of 37 Bacteria from the Human Vaginal Tract. Microbiol Resour Announc 12(6): e0035823. 10.1128/mra.00358-23.
9. Edward EA, Mohamed NM, and Zakaria AS. (2022) Whole Genome Characterization of the High-Risk Clone ST383 *Klebsiella pneumoniae* with a Simultaneous Carriage of *bla*(CTX-M-14) on IncL/M Plasmid and *bla*(CTX-M-15) on Convergent IncHI1B/IncFIB Plasmid from Egypt. Microorganisms 10(6). 10.3390/microorganisms10061097.
10. Turton JF, Payne Z, Coward A, Hopkins KL, Turton JA, Doumith M, and Woodford N. (2018) Virulence genes in isolates of *Klebsiella pneumoniae* from the UK during 2016, including among carbapenemase gene-positive hypervirulent K1-ST23 and 'non-hypervirulent' types ST147, ST15 and ST383. J Med Microbiol 67(1): 118-128. 10.1099/jmm.0.000653.
11. Turton J, Davies F, Turton J, Perry C, Payne Z, and Pike R. (2019) Hybrid Resistance and Virulence Plasmids in "High-Risk" Clones of *Klebsiella pneumoniae*, Including Those Carrying *bla*(NDM-5). Microorganisms 7(9). 10.3390/microorganisms7090326.

12. Starkova P, Lazareva I, Avdeeva A, Sulian O, Likholetova D, Ageevets V, Lebedeva M, Gostev V, Sopova J, and Sidorenko S. (2021) Emergence of Hybrid Resistance and Virulence Plasmids Harboring New Delhi Metallo-β-Lactamase in *Klebsiella pneumoniae* in Russia. Antibiotics (Basel) 10(6). 10.3390/antibiotics10060691.
13. Falcone M, Tiseo G, Arcari G, Leonildi A, Giordano C, Tempini S, Bibbolino G, Mozzo R, Barnini S, Carattoli A, and Menichetti F. (2022) Spread of hypervirulent multidrug-resistant ST147 *Klebsiella pneumoniae* in patients with severe COVID-19: an observational study from Italy, 2020-21. J Antimicrob Chemother 77(4): 1140-1145. 10.1093/jac/dkab495.

Supplementary Figure S1.

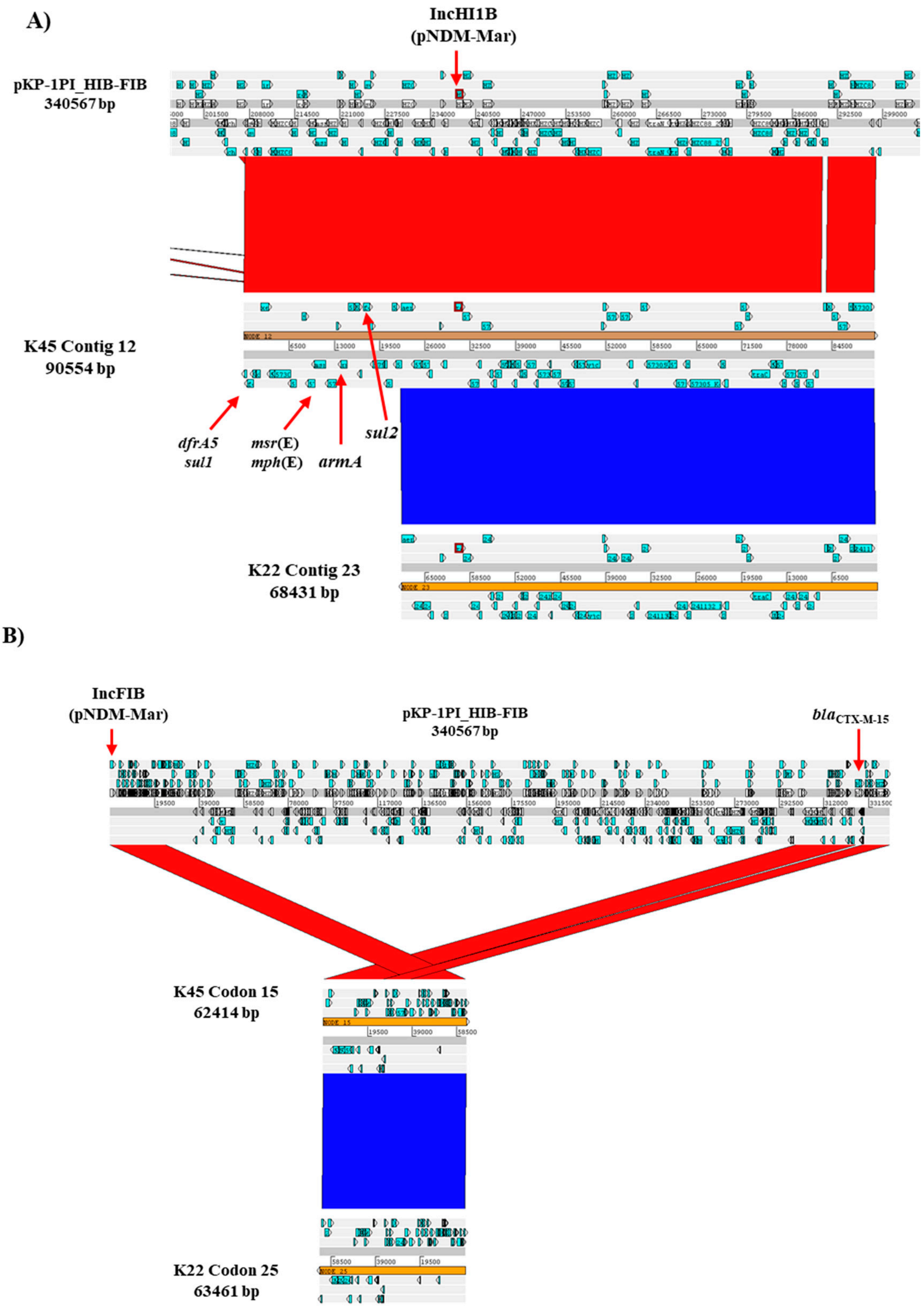

Supplementary Figure S1 (Continued).

C)

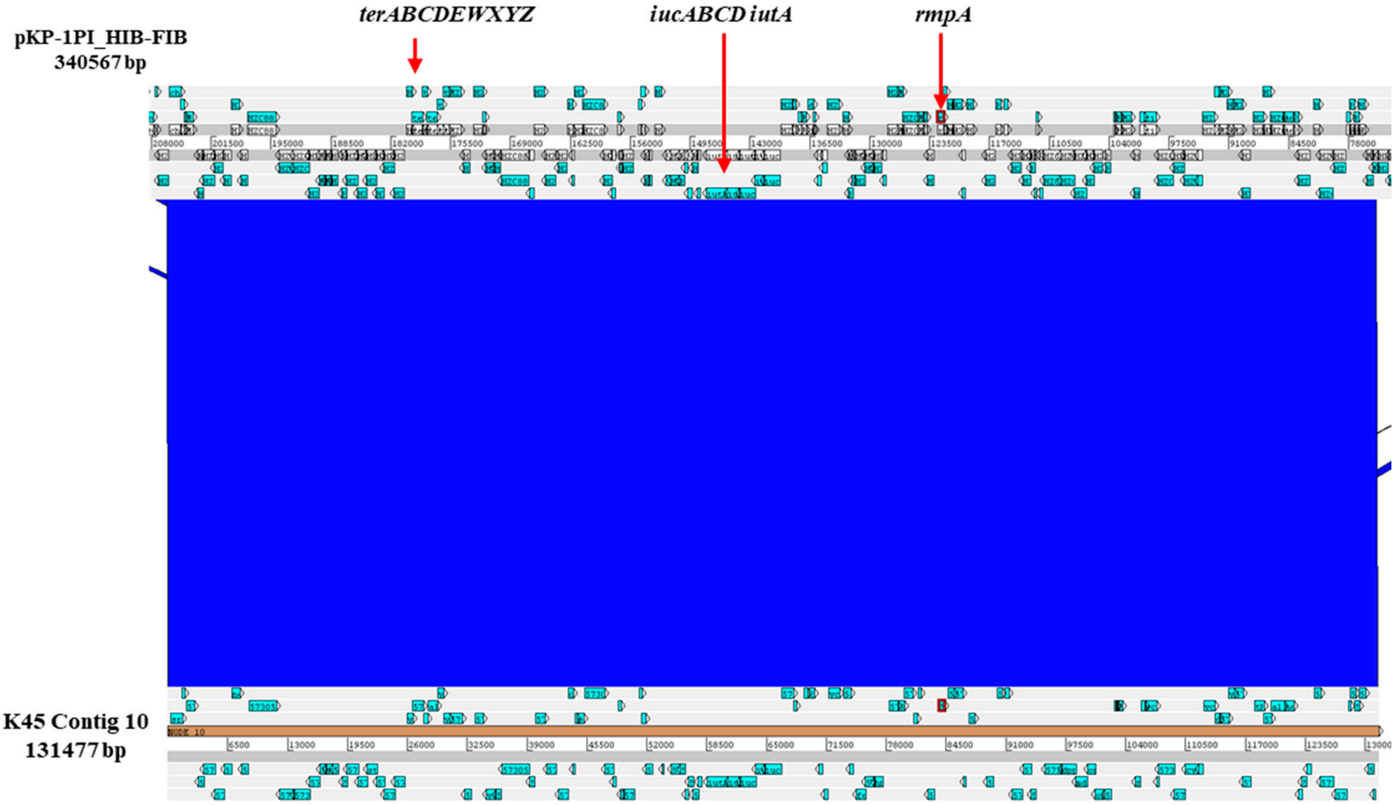

Supplementary Figure S2.

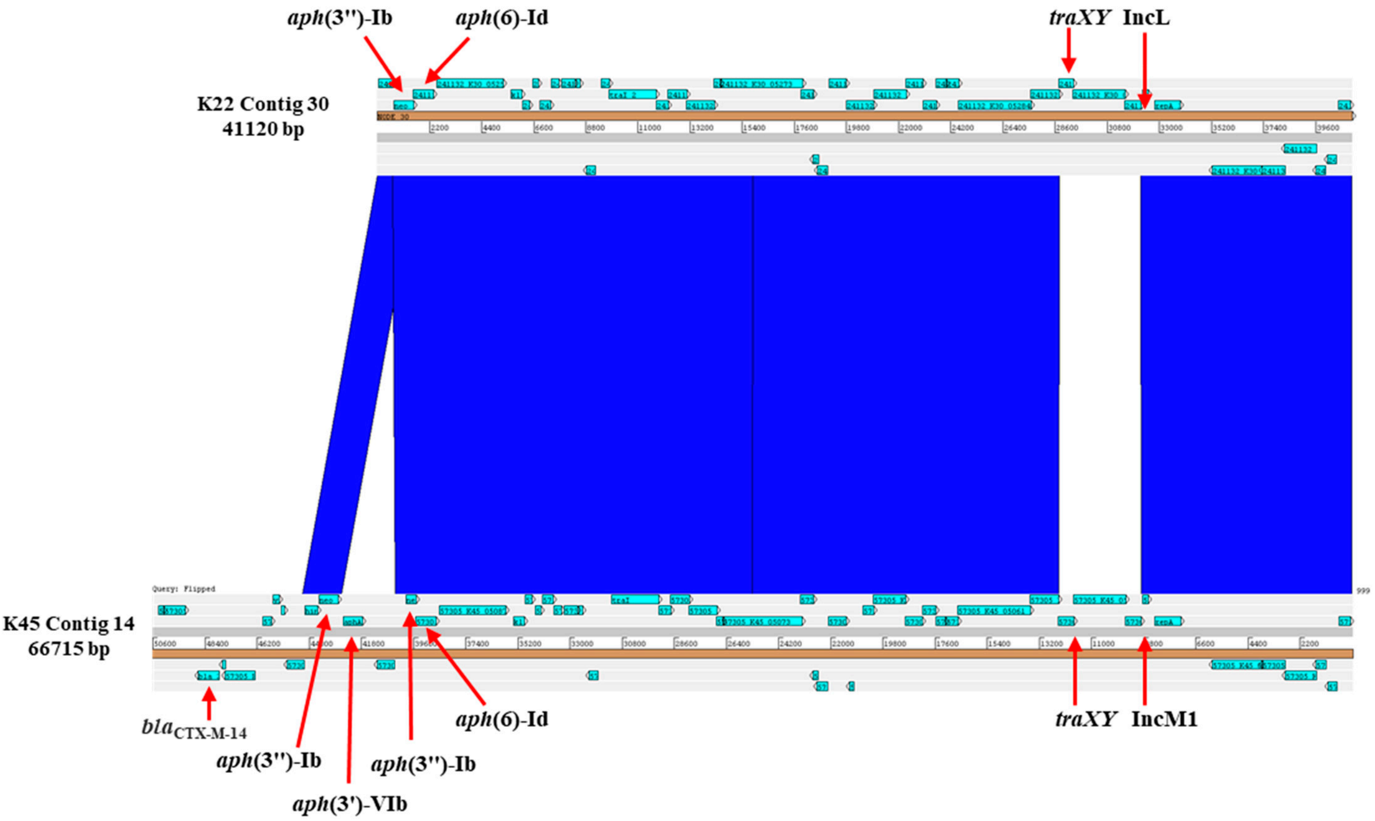

Supplementary Figure S3.

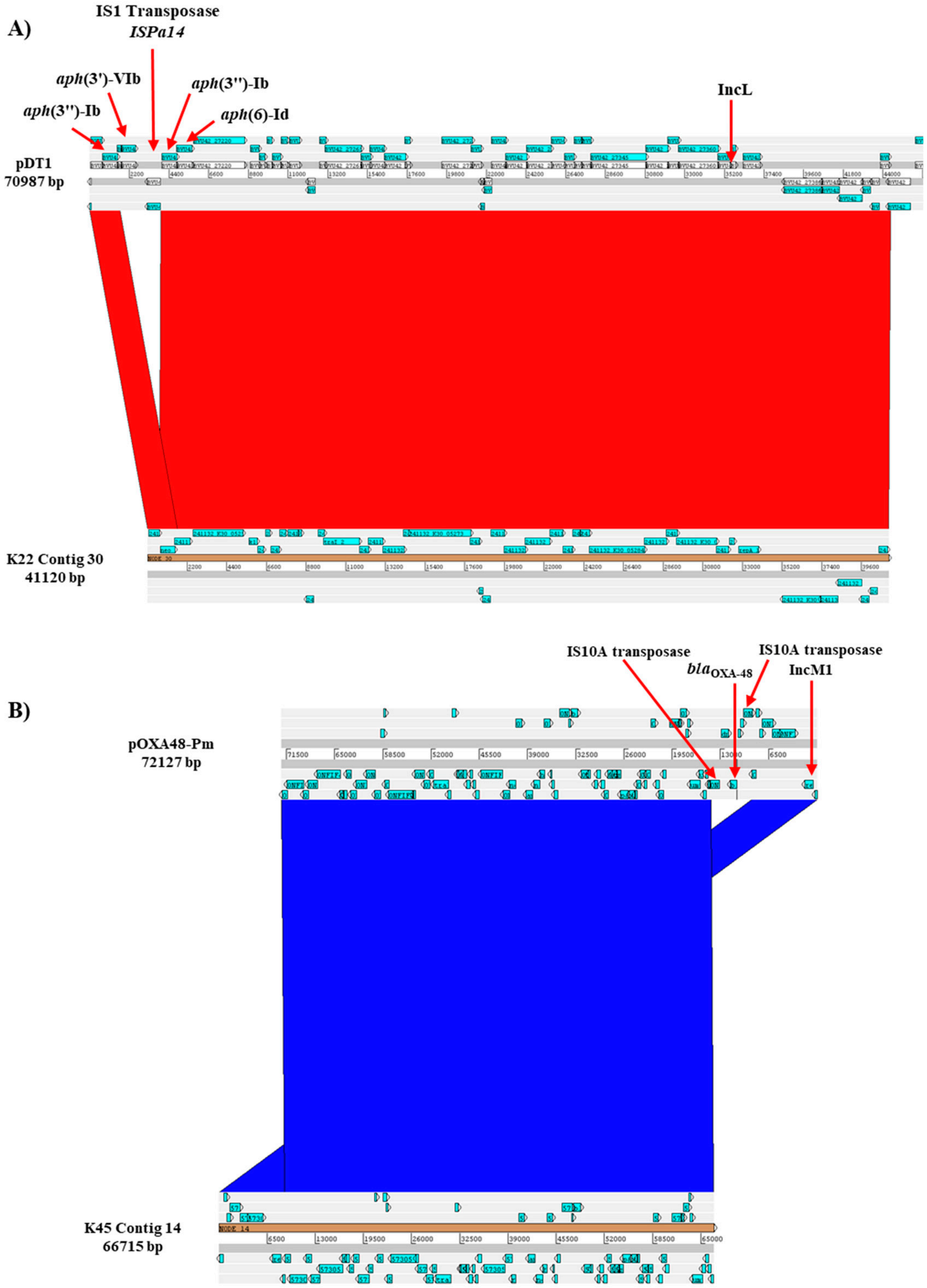

Supplementary Figure S4.

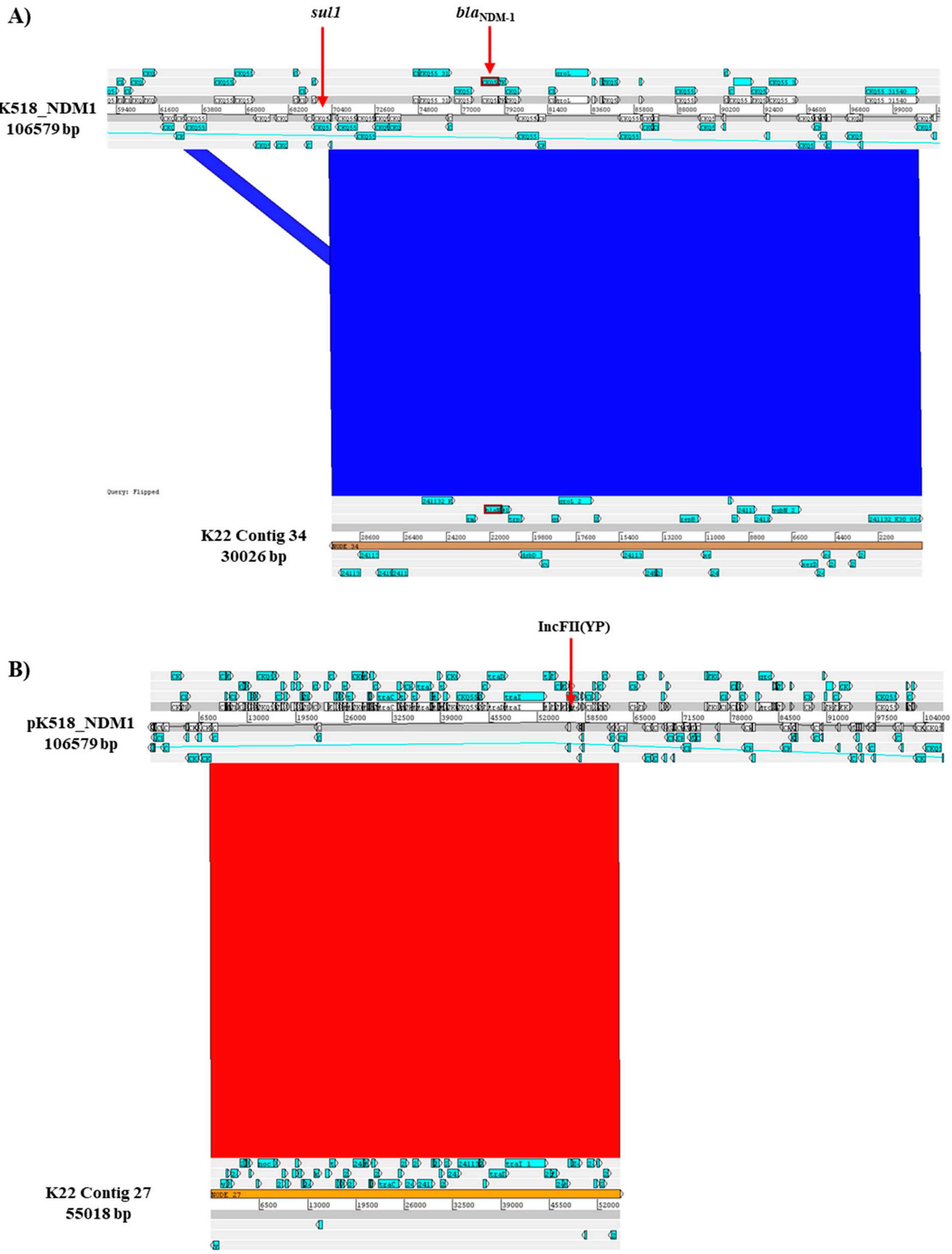

Supplementary Figure S5.

A)

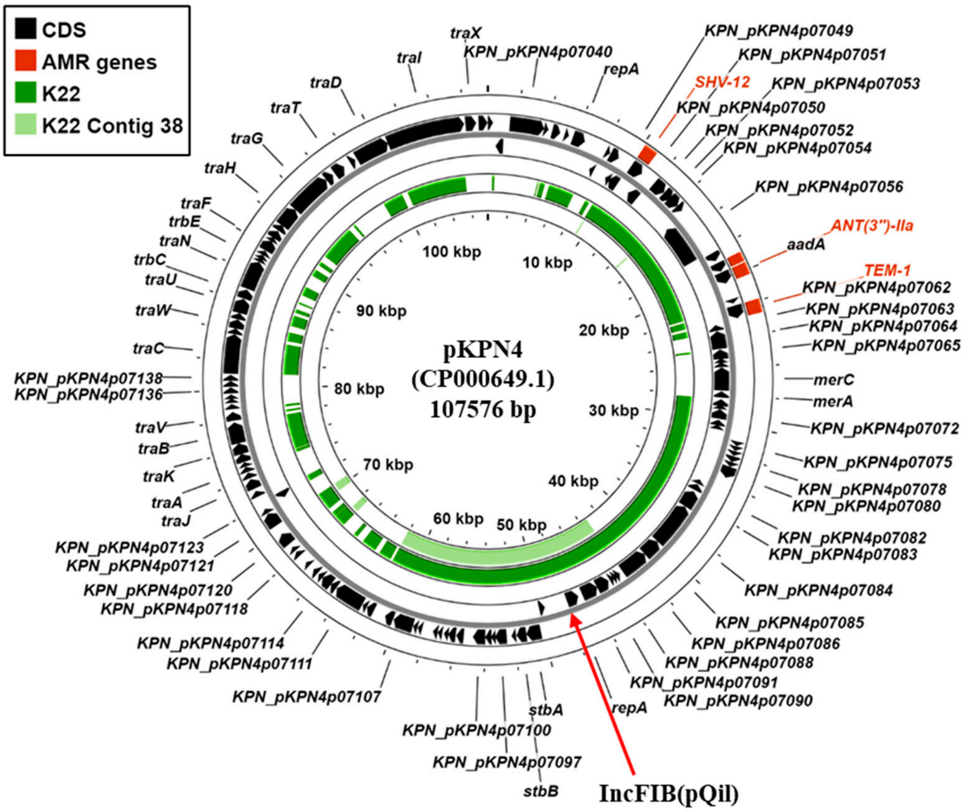

B)

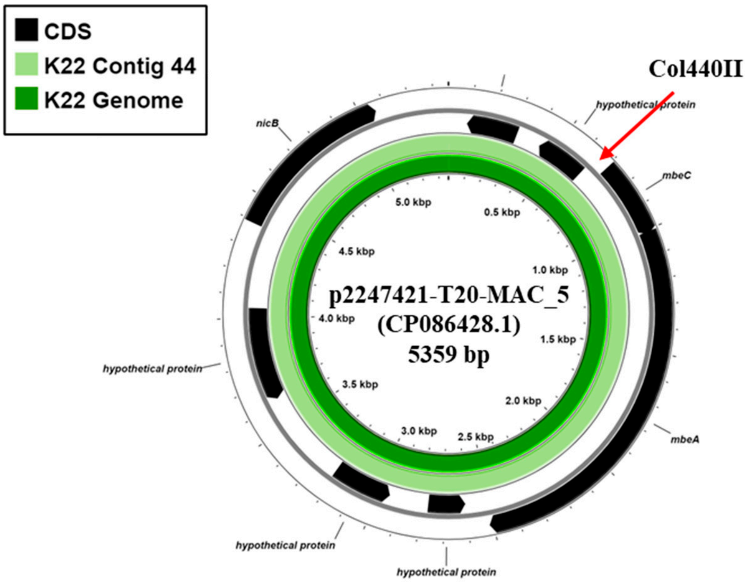

Supplementary Figure S6.

A)

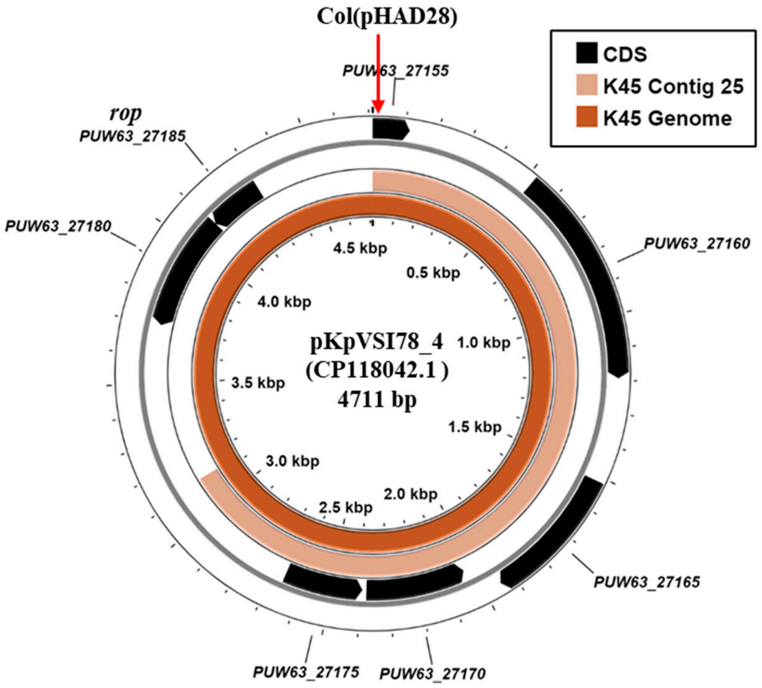

B)

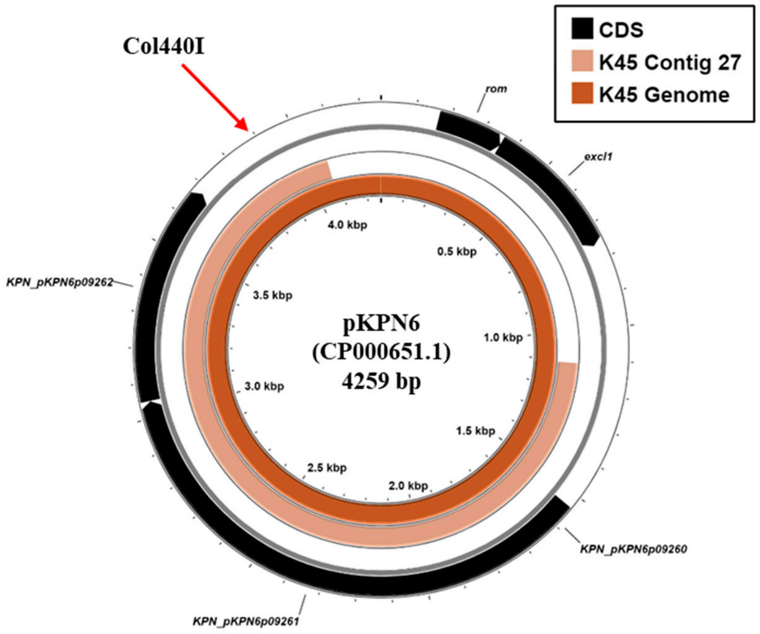

Supplementary Figure S7.

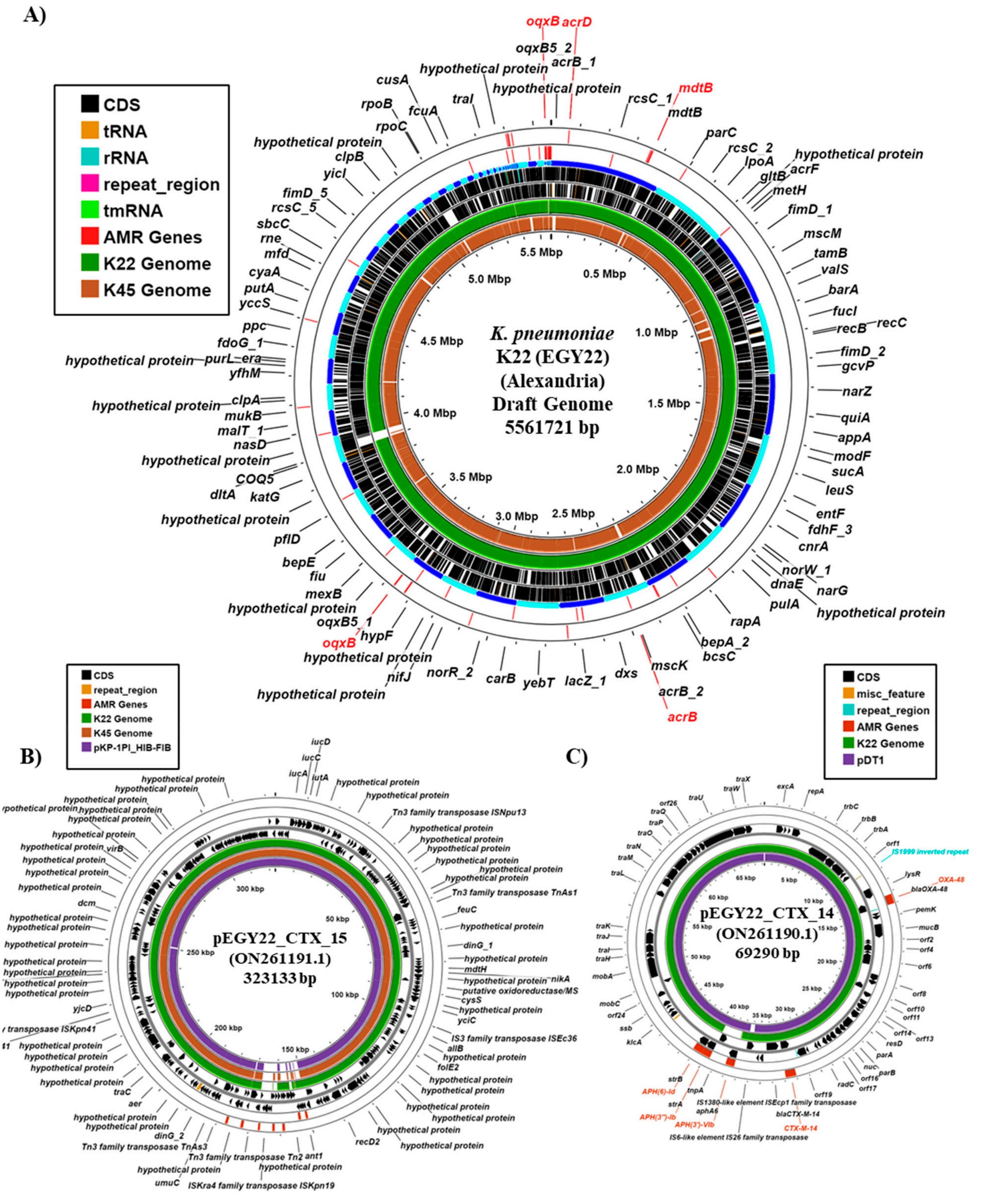

Supplementary Figure S8.

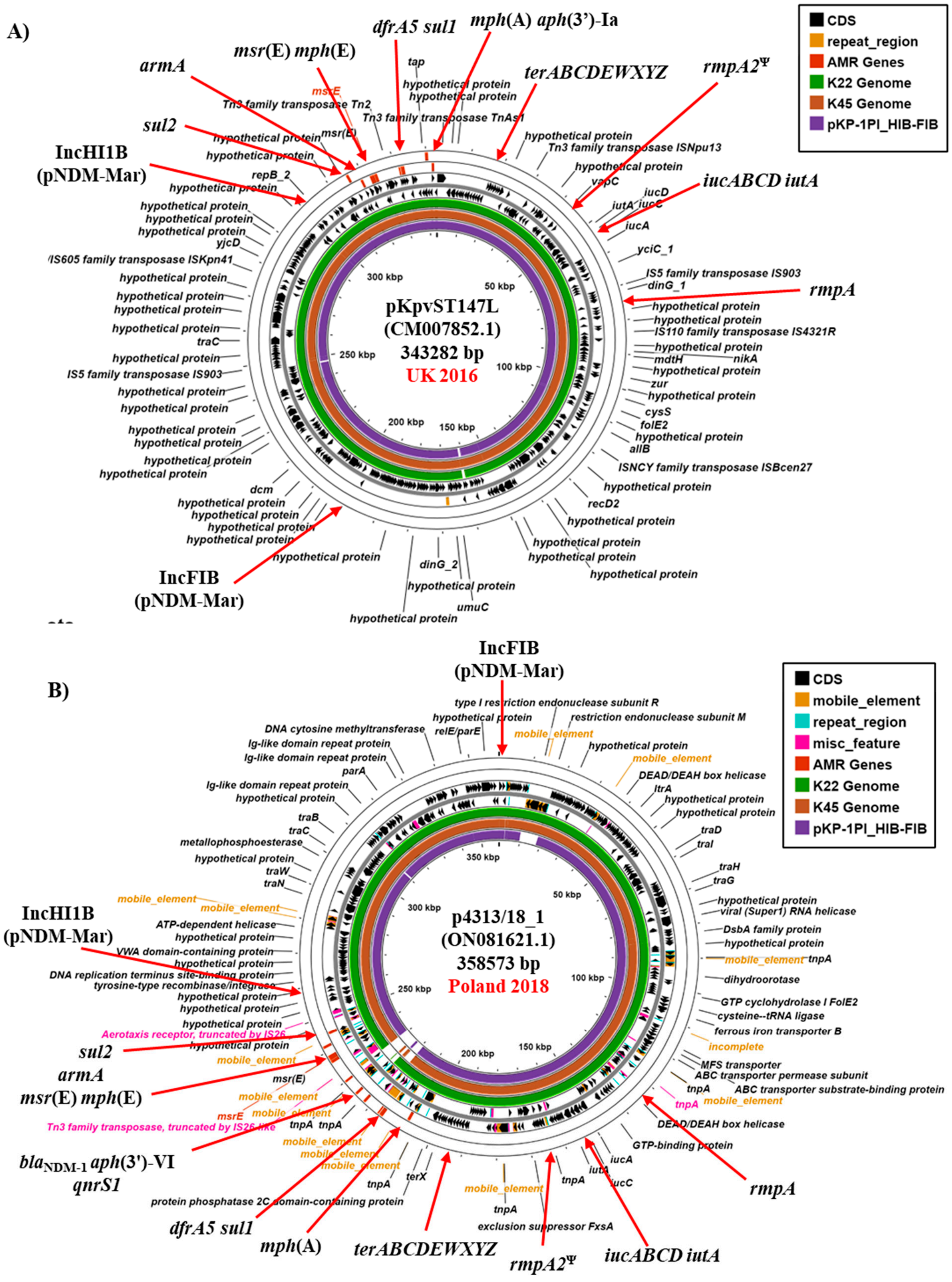

Supplementary Figure S8 (continued).

C)

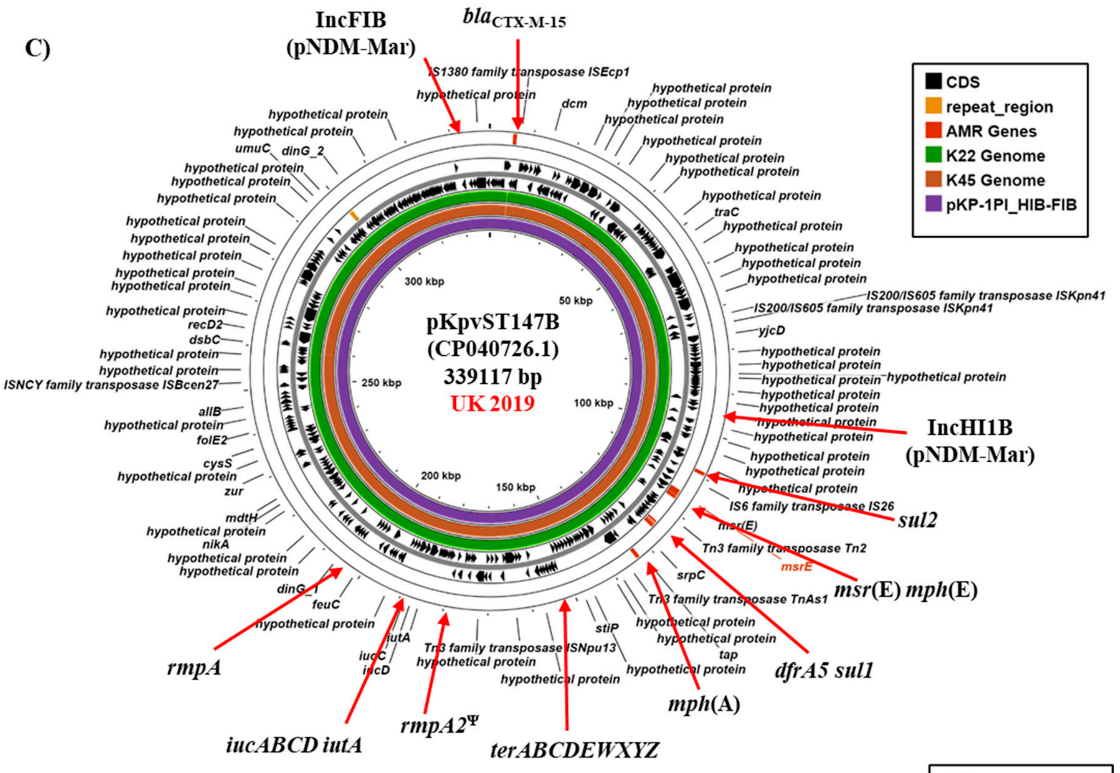

D)

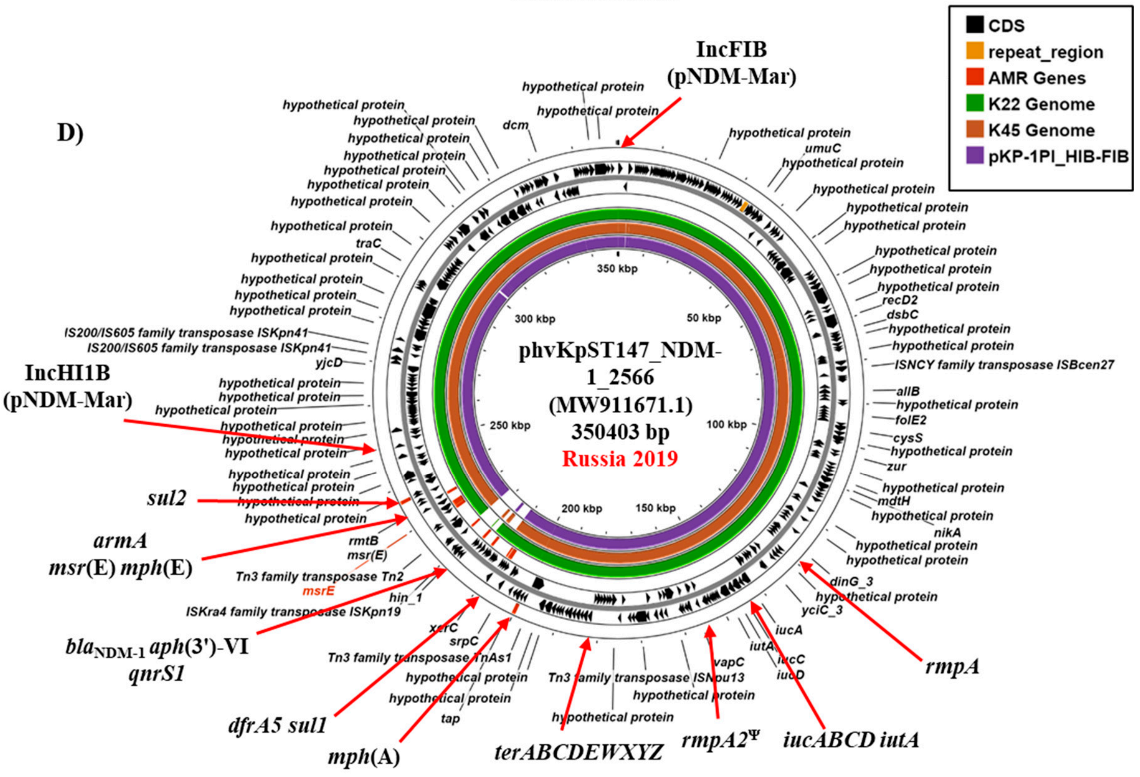

Supplementary Figure S8 (continued).

E)

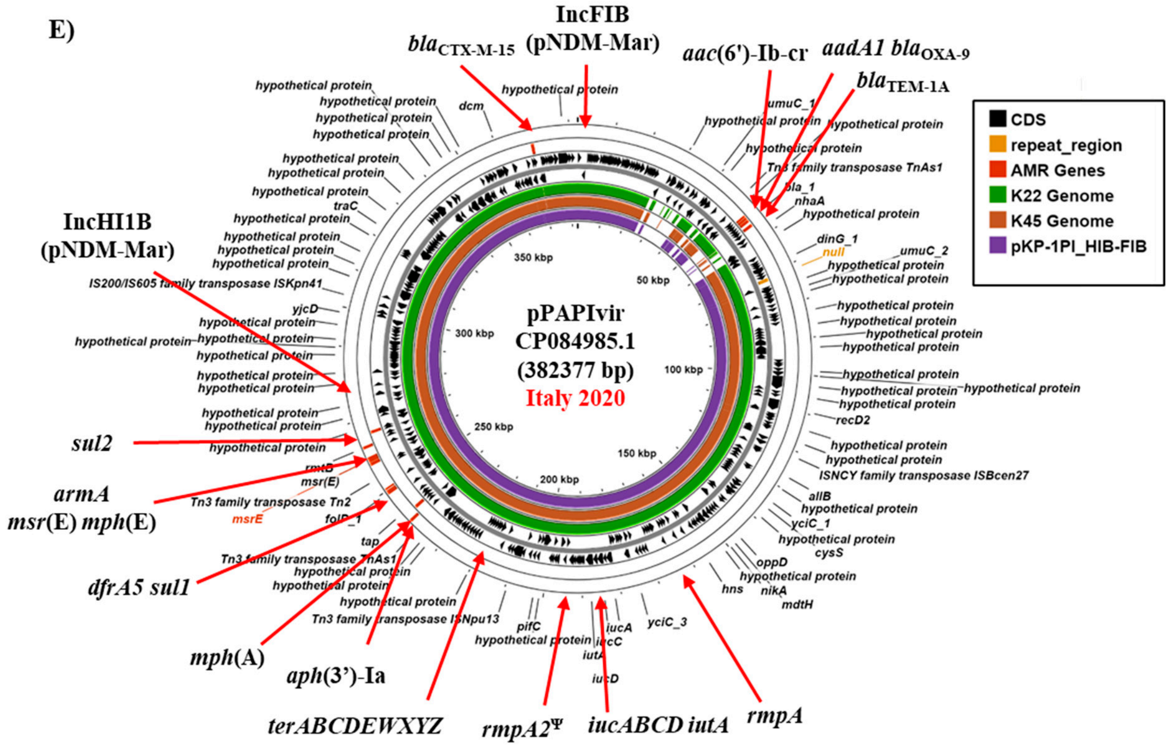

Supplement: Supplementary file 1 [file microorganisms-13-01058-s001.zip › microorganisms-3578253-supplementary.pdf]
